# A composite index; socioeconomic deprivation and coverage of reproductive and maternal health interventions

**DOI:** 10.2471/BLT.23.290866

**Published:** 2023-12-08

**Authors:** Leonardo Z Ferreira, Fernando C Wehrmeister, Jakob Dirksen, Luis Paulo Vidaletti, Monica Pinilla-Roncancio, Katherine Kirkby, Luiza IC Ricardo, Aluisio JD Barros, Ahmad Reza Hosseinpoor

**Affiliations:** a International Center for Equity in Health, Universidade Federal de Pelotas, R. Marechal Deodoro 1160, Centro, Pelotas, Brazil.; b Oxford Poverty and Human Development Initiative, University of Oxford, Oxford, England.; c Centre of Sustainable Development Goals for Latin America and the Caribbean and School of Medicine, Universidad de los Andes, Bogotá, Colombia.; d Department of Data and Analytics, World Health Organization, Geneva, Switzerland.

## Abstract

**Objective:**

To examine inequalities in the coverage of reproductive and maternal health interventions in low- and middle-income countries and territories using a composite index of socioeconomic deprivation status.

**Methods:**

We obtained data on education and living standards from national household surveys conducted between 2015 and 2019 to calculate socioeconomic deprivation status. We assessed the coverage of reproductive and maternal health interventions, using three indicators: (i) demand for family planning satisfied with modern methods; (ii) women who received antenatal care in at least four visits; and (iii) the presence of a skilled attendant at delivery. Absolute and relative inequalities were evaluated both directly and using the slope index of inequality and the concentration index.

**Findings:**

In the 73 countries and territories with available data, the median proportions of deprivation were 41% in the low-income category, 11% in the lower-middle-income category and less than 1% in the upper-middle-income category. The coverage analysis, conducted for 48 countries with sufficient data, showed consistently lower median coverage among deprived households across all health indicators. The coverage of skilled attendant at delivery showed the largest inequalities, where coverage among the socioeconomically deprived was substantially lower in almost all countries. Antenatal care visits and demand for family planning satisfied with modern methods also showed significant disparities, favouring the less deprived population.

**Conclusion:**

The findings highlight persistent disparities in the coverage of reproductive and maternal health interventions, requiring efforts to reduce those disparities and improve coverage, particularly for skilled attendant at delivery.

## Résumé

**Objectif:**

Examiner les inégalités dans la couverture des interventions de santé reproductive et maternelle dans les pays et territoires à revenu faible et intermédiaire à l’aide d’un indice composite du statut de privation socioéconomique.

**Méthodes:**

Nous avons obtenu des données sur l’éducation et les niveaux de vie à partir d’enquêtes nationales sur les ménages menées entre 2015 et 2019 pour calculer le statut de privation socioéconomique. Nous avons évalué la couverture des interventions de santé reproductive et maternelle à l’aide de trois indicateurs: (i) une demande de planification familiale satisfaite avec des méthodes modernes; (ii) le fait que les femmes reçoivent des soins prénatals lors d’au moins quatre visites; et (iii) la présence de personnel qualifié présent lors de l’accouchement. Les inégalités absolues et relatives ont été évaluées à la fois directement et à l’aide de l’indice de pente et l’indice de concentration des inégalités.

**Résultats:**

Dans les 73 pays et territoires pour lesquels des données sont disponibles, les proportions médianes de privation étaient de 41% dans la catégorie à faibles revenus, de 11% dans la catégorie à revenus moyens inférieurs et de moins de 1% dans la catégorie des revenus moyens supérieurs. L’analyse de cette couverture, menée dans 48 pays disposant de données suffisantes, a mis en évidence que la couverture médiane était systématiquement plus faible dans les ménages défavorisés pour tous les indicateurs de santé. La couverture de personnel qualifié présent lors de l’accouchement est celle qui présente les inégalités les plus importantes, le chiffre pour les ménages défavorisés étant nettement inférieur dans presque tous les pays. Les visites de soins prénatals et la demande en planning familial satisfait par des méthodes modernes ont également indiqué des disparités considérables, en faveur des populations moins démunies.

**Conclusion:**

Les résultats mettent en évidence des disparités persistantes dans la couverture des interventions de santé reproductive et maternelle, ce qui nécessite des efforts pour réduire ces disparités et améliorer la couverture, en particulier en ce qui concerne l’accouchement assisté par du personnel qualifié.

## Resumen

**Objetivo:**

Estudiar las desigualdades en la cobertura de las intervenciones de salud reproductiva y materna en países y territorios de ingresos bajos y medios mediante un índice compuesto del nivel de privación socioeconómica.

**Métodos:**

Se obtuvieron datos sobre el nivel educativo y de vida a partir de encuestas nacionales de hogares realizadas entre 2015 y 2019 para calcular el estado de privación socioeconómica. Se evaluó la cobertura de las intervenciones de salud reproductiva y materna mediante tres indicadores: (i) demanda de planificación familiar satisfecha con métodos modernos; (ii) mujeres que recibieron atención prenatal en al menos cuatro visitas; y (iii) presencia de personal calificado para atender partos. Las desigualdades absolutas y relativas se evaluaron directamente y mediante el índice de pendiente de la desigualdad y el índice de concentración.

**Resultados:**

En los 73 países y territorios con datos disponibles, los porcentajes medios de privación eran del 41% en la categoría de ingresos bajos, del 11% en la de ingresos medios-bajos y de menos del 1% en la de ingresos medios-altos. El análisis de la cobertura, que se realizó en 48 países con datos suficientes, mostró una cobertura mediana sistemáticamente inferior entre los hogares desfavorecidos en todos los indicadores de salud. La cobertura de personal calificado para atender partos mostró las mayores desigualdades, ya que la cobertura entre las personas socioeconómicamente desfavorecidas fue sustancialmente inferior en casi todos los países. Las visitas de atención prenatal y la demanda de planificación familiar satisfecha con métodos modernos también mostraron diferencias significativas, favoreciendo a la población menos desfavorecida.

**Conclusión:**

Los resultados evidencian la persistencia de diferencias en la cobertura de las intervenciones de salud reproductiva y materna, lo que exige esfuerzos para reducir esas diferencias y mejorar la cobertura, en especial de personal calificado para la atención de partos.

## ملخص

الغرض دراسة التفاوتات في تغطية تدخلات الصحة الإنجابية وصحة الأم في الدول والأقاليم ذات الدخل المنخفض والدخل المتوسط، وذلك باستخدام مؤشر مركب لحالة الحرمان الاجتماعي والاقتصادي.

الطريقة حصلنا على بيانات حول مستويات التعليم والمعيشة من المسوحات الأسرية الوطنية، والتي أُجريت بين عامي 2015 و2019 لتقدير حالة الحرمان الاجتماعي والاقتصادي. قمنا بتقييم تغطية التدخلات المتعلقة بالصحة الإنجابية وصحة الأم، وذلك باستخدام ثلاثة مؤشرات: (1) تلبية الطلب على تنظيم الأسرة بالطرق الحديثة؛ و(2) النساء اللاتي حصلن على رعاية ما قبل الولادة في أربع زيارات على الأقل؛ و(3) وجود مساعدة ماهرة عند الولادة. تم تقييم التفاوتات المطلقة والنسبية سواء بشكل مباشر أو باستخدام مؤشر المنحدر للتفاوت، ومؤشر التركيز.

النتائج في 73 دولة وإقليمًا ذات بيانات متوفرة، كان متوسط نسب الحرمان %41 في فئة الدخل المنخفض، و%11 في فئة الدخل المتوسط الأدنى، وأقل من %1 في فئة الدخل المتوسط الأعلى. وبالنسبة لتحليل التغطية، الذي أُجري في 48 دولة ذات بيانات كافية، فقد أظهر انخفاضًا مستمرًا في متوسط التغطية بين الأسر المحرومة في جميع المؤشرات الصحية. وأظهرت التغطية بواسطة قابلة ماهرة عند الولادة أكبر التفاوتات، حيث كانت التغطية بين المحرومين اجتماعيا واقتصاديا أقل بشكل ملموس في جميع الدول تقريبًا. كما أن زيارات الرعاية قبل الولادة، والطلب على تنظيم الأسرة الذي يتم تلبيته بالطرق الحديثة، أظهرت تفاوتات ملموسة، لصالح السكان الأقل حرمانًا.

الاستنتاج تسلط النتائج الضوء على التفاوتات الدائمة في تغطية تدخلات الصحة الإنجابية وصحة الأم، مما يتطلب بذل جهود للحد من تلك التفاوتات، وتحسين التغطية، وخاصة بالنسبة للقابلات الماهرات عند الولادة.

## 摘要

**目的:**

利用社会经济贫困状况的综合指数，评估低收入和中等收入国家和地区在生育和孕产妇健康干预措施覆盖率方面的不平等现象**。**

**方法:**

我们从 2015 年至 2019 年期间进行的全国家庭调查中获得了有关教育和生活水平的数据，以推测社会经济贫困状况。我们使用三个指标评估了生育和孕产妇健康干预措施的覆盖率：(i) 使用现代方法满足计划生育需求；(ii) 至少接受过四次产前护理的妇女；(iii) 分娩时有熟练的助产士在场。直接评估和使用不平等斜率指数和集中指数评估绝对不平等和相对不平等状况。

**结果:**

在有数据可查的 73 个国家和地区中，低收入类别国家的贫困比例中位数为 41%，中低收入类别国家为 11%，而中高收入类别国家为不到 1%。针对 48 个拥有充足数据的国家进行的覆盖率分析显示，在所有健康指标中，贫困家庭的覆盖率中位数一直较低。分娩时熟练助产士的覆盖率显示出的不平等情况最为严重，在几乎所有国家的社会经济贫困人口的覆盖率都明显低很多。接受产前护理和使用现代方法满足计划生育需求的情况也显示出了显著的差异，可以帮助贫困程度较低的人群。

**结论:**

研究结果高度表明了在生育和孕产妇健康干预措施的覆盖率方面仍然存在差距，需要努力缩小这些差距并提高覆盖率，特别是熟练助产士的覆盖率。

## Резюме

**Цель:**

Изучить неравенство в охвате мероприятий по охране репродуктивного здоровья и здоровья матери в странах и территориях с низким и средним уровнем дохода с помощью обобщенного показателя статуса социально-экономического неравенства.

**Методы:**

Для расчета статуса социально-экономического неравенства были получены данные об образовании и уровне жизни из национальных обследований домашних хозяйств, проведенных в период с 2015 по 2019 год. Оценка охвата мероприятиями по охране репродуктивного здоровья и здоровья матери проводилась на основании трех показателей: (i) удовлетворение спроса на планирование семьи современными методами; (ii) получение женщинами дородового наблюдения в ходе не менее четырех визитов; (iii) присутствие квалифицированного акушера при родах. Абсолютное и относительное неравенство оценивалось как непосредственно, так и с помощью индекса снижения неравенства и индекса концентрации.

**Результаты:**

В 73 странах и территориях, по которым доступны данные, медианная доля неравенства составила 41% в категории с низким уровнем дохода, 11% в категории с уровнем дохода ниже среднего и менее 1% в категории с уровнем дохода выше среднего. Результаты анализа охвата, проведенного по 48 странам с достаточным объемом данных, показали неизменно более низкий медианный охват среди социально неблагополучных домашних хозяйств по всем показателям здоровья. Охват квалифицированными акушерами во время родов продемонстрировал наибольшее неравенство: почти во всех странах охват среди социально и экономически неблагополучных слоев населения был значительно ниже. Визиты для дородового наблюдения и спрос на услуги по планированию семьи с использованием современных методов также продемонстрировали значительные диспропорции в пользу более обеспеченных слоев населения.

**Вывод:**

Полученные данные свидетельствуют о сохраняющемся неравенстве в охвате мероприятий по охране репродуктивного здоровья и здоровья матери, что требует усилий по сокращению этого неравенства и расширению охвата, особенно в отношении квалифицированной помощи при родах.

## Introduction

Eliminating poverty in all its forms is the first of the 17 sustainable development goals (SDGs).^1^ People living in conditions of poverty are likely to have fewer opportunities for health care, thus experiencing more adverse health outcomes and a higher risk of preventable and premature death.^2^ Furthermore, inequities in the coverage of reproductive, maternal and child health interventions correlate strongly with disparities in maternal and child mortality.^3^^,^^4^ Hence, addressing these inequities is important not only for reducing maternal and child mortality rates, but also for the advancement of SDG 3, which aims to ensure healthy lives and promote well-being for all.^1^ Monitoring and analysing inequalities across various dimensions, such as subnational regions and poverty levels is essential for tracking progress towards the SDGs, aimed at leaving no one behind. Understanding these disparities is key to identifying strategies and policies that can break vicious cycles of ill-health and poverty.^5^

Applying an equity lens is particularly important in low- and middle-income countries, which are home to the largest share and number of people living in conditions of poverty.^6^ Most of these countries rely on national household surveys as their primary source of information for reproductive, maternal and child health. Traditionally, socioeconomic inequalities have been assessed in these surveys by using a combination of assets (such as mobile phones, refrigerators and bicycles), basic services and household materials to generate a score to rank households’ wealth.^7^ This score, or wealth index, is often divided into five equally sized groups and presented as wealth quintiles. Despite its widespread application, the wealth index has several well-documented limitations.^8^^–^^10^ The aim of the index is to provide a relative wealth distribution for the country at the time of the survey. Hence, its design affects the suitability for cross-country comparisons and broader comparisons over time. The index tends to favour urban populations, a bias partly influenced by better infrastructure and higher asset ownership in urban areas. Furthermore, the index, based on a single dimension of socioeconomic position, presents a different picture from consumption or expenditure. Recently, we proposed a multidimensional measure of socioeconomic deprivation, integrating education and material living standards, inspired by the United Nations (UN) Multidimensional Poverty Index.^6^^,^^8^ The new measure benefits from being an absolute measure of deprivation with fixed cut-offs, unlike the wealth index score, which changes from country to country, and even from survey to survey within the same country. As with the wealth index, the new socioeconomic deprivation status can be calculated using data from national health surveys.

Using this new measure of socioeconomic deprivation status, we conducted a comprehensive analysis of the distribution of socioeconomic deprivation across 73 low- and middle-income countries and territories. We subsequently describe and compare reproductive and maternal health inequalities for those countries with significant levels of deprivation, using the measure of socioeconomic deprivation status. The findings establish a baseline for monitoring progress in increasing coverage and reducing inequalities in essential health indicators.

## Methods

To calculate the composite index of socioeconomic deprivation status, we obtained data from household surveys conducted by the Demographic and Health Surveys^11^ and the Multiple Indicator Cluster Survey^12^ programmes. These surveys are designed to be nationally representative by employing a multistage sampling framework, that often uses census tracts as the primary sampling unit. In April 2022, we selected the most recent surveys conducted after 2015 that had available data for the calculation of the socioeconomic deprivation status measure from all low- and middle-income countries and territories. 

We calculated the socioeconomic deprivation status by using eight indicators across the two dimensions, education and living standards (Table 1). The educational indicators cover school attendance of school-aged children, and years of schooling for household members older than 10 years. The living standards indicators cover the type of fuel used for cooking, access to improved sanitation facilities, a safe source of drinking water and electricity, as well as assets ownership and adequate housing materials. Each indicator is coded as 1 for deprived households and 0 for non-deprived households. We then combined these indicators to generate a composite deprivation score, whereby each dimension accounts for 50% of the total weight, with each indicator having equal weights within each dimension. The composite score ranges from 0 to 1, with higher values indicating more severe status of socioeconomic deprivation of a household and its members. Based on the original cut-offs, the score intervals were broken down into three groups: not deprived (score ranging from 0 to 0.25); vulnerable (score ranging from above 0.25 to 0.50); and deprived (score above 0.50). In this study, the deprived group combines the deprived and extremely deprived categories from the original socioeconomic deprivation status,^6^ as the sample size for the extremely deprived group was too small (below 25 households) for meaningful analyses. 

**Table 1:** Structure of the socioeconomic deprivation status index

Dimensions of disadvantage and indicator	Deprived if…	Weight
**Education**		
Years of schooling	No eligible household member has completed at least 6 years of schooling	1/4
School attendance	Any school-aged child is not attending school up to the age at which they would complete class 8	1/4
**Living standards**		
Cooking fuel	A household cooks using solid fuel, such as dung, agricultural crop, shrubs, wood, charcoal or coal	1/12
Sanitation	The household has unimproved or no sanitation facility, or it is improved but shared with other households	1/12
Drinking water	The household’s source of drinking water is not safe, or safe drinking water is a 30-minute or longer walk from home, roundtrip	1/12
Electricity	The household has no electricity	1/12
Housing	The household has inadequate housing materials in any of the three components: floor, roof or walls	1/12
Assets	The household does not own more than one of these assets: radio, television, telephone, computer, animal cart, bicycle, motorbike or refrigerator, and does not own a car or truck	1/12

Source: Dirksen et al.^6^

To examine how the level of socioeconomic deprivation status related to coverage of reproductive and maternal health interventions, we selected commonly used key indicators to monitor these interventions. Furthermore, these indicators have large sample sizes in the surveys used. We generated estimates for three indicators covering the reproductive, pregnancy and birth stages: (i) demand for family planning satisfied with modern methods; (ii) women who received antenatal care in at least four visits; and (iii) the presence of a skilled attendant at delivery. Detailed definitions of all indicators can be found in Table 2. 

**Table 2:** Definition of three reproductive and maternal health indicators used to measure inequalities in reproductive and maternal health

Indicator	Numerator	Denominator
Demand for family planning satisfied by modern methods	Who is using (or whose partner is using) a modern contraceptive method	Women aged 15–49 years either married or in union in need of contraception
Antenatal care four or more visits	Received at least four antenatal care visits with any provider	Women aged 15–49 years who had a birth in the past 3 years before the survey
Skilled attendant at delivery	Delivered by a skilled attendant (based on each country’s definition of skilled attendant)	Women aged 15–49 years who had a birth in the past 3 years before the survey

We disaggregated coverage estimates for these indicators by the socioeconomic deprivation status. We visually evaluated equity patterns according to the inverse equity hypothesis.^13^ This hypothesis proposes that health coverage first increases in wealthier populations. As a result, absolute health inequalities are expected to widen initially, only diminishing when these interventions eventually reach the more deprived groups. Hence, upon introducing new health interventions, top inequality patterns often emerge, where the most privileged group (that is, the non-deprived group) benefits, leaving other groups behind. As the interventions reach more people, bottom inequality patterns usually arise when the most deprived group considerably lags behind.^13^ When the gaps between the groups are of similar magnitude, it is referred to as linear inequality. We assessed absolute and relative health inequalities using the slope index of inequality and the concentration index, respectively. Both measures range from −100 to +100, where negative values indicate higher coverage for the most disadvantaged, positive values indicate higher coverage for the households with less deprivation, and zero indicates absence of inequality.^14^

We restricted the coverage analyses to countries with sufficient sample sizes (at least 25 households) for the socioeconomic deprivation status. Results are presented by country or territory, and grouped according to the World Bank’s country income level classification^15^ for the year the survey was completed. We conducted all analyses using Stata version 17 (StataCorp. LP, College Station, United States of America), accounting for sampling weights and the clustered design of the surveys. Ethical clearance was obtained by the United States Agency for International Development and the United Nations Children’s Fund, which funded the data collection.

## Results

**Fig. 1:**
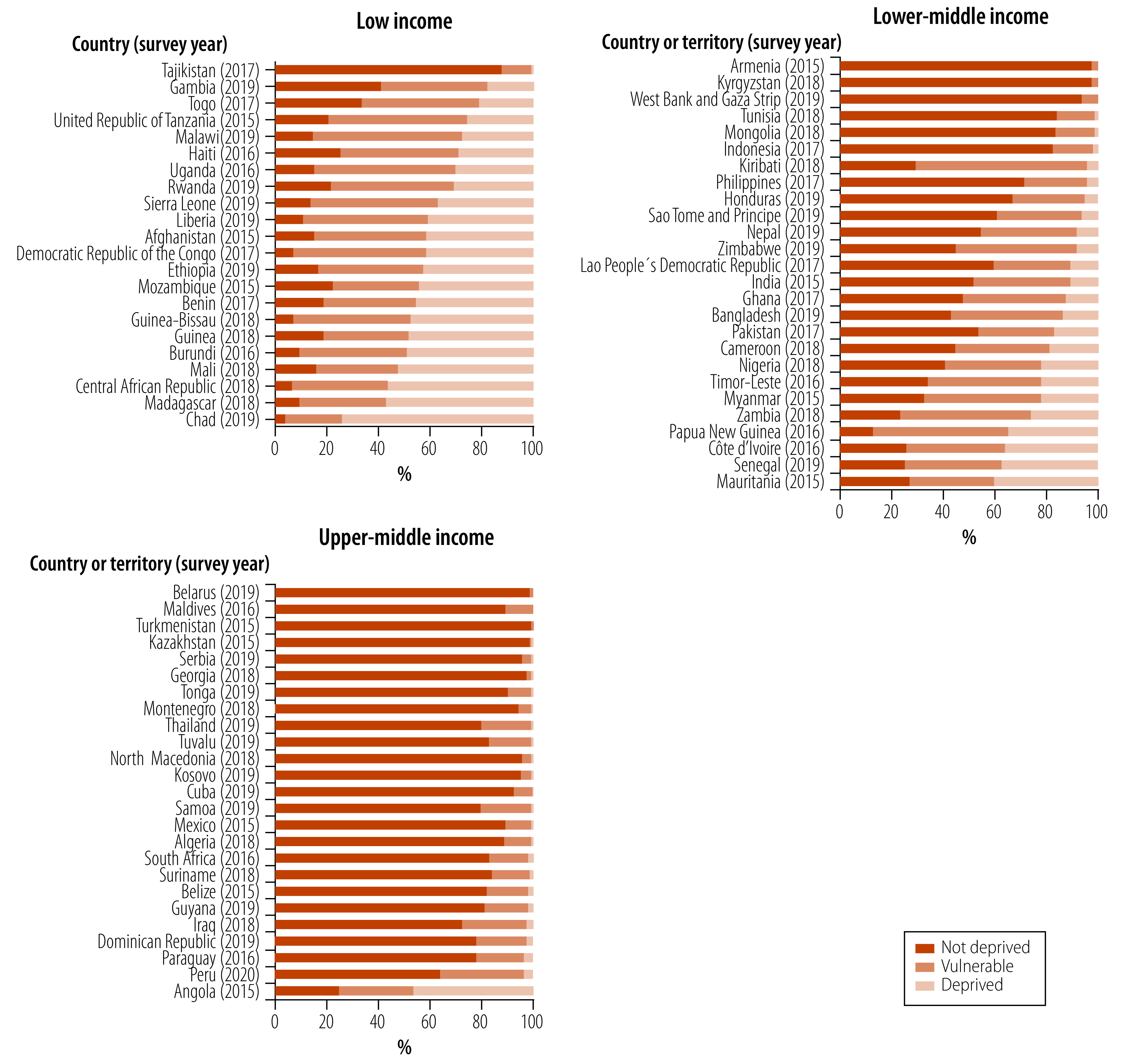
The distribution of the socioeconomic deprivation status for all countries and territories considered for the analysis by World Bank income level classification

A total of 73 countries and territories were eligible for the study, comprising 22 low-income, 26 lower-middle-income and 25 upper-middle-income countries and territories (Table 3). The distribution of the socioeconomic deprivation status in each country or territory is presented in Fig. 1. In half (25) of the low- and lower-middle-income countries and territories, over a quarter of the population was classified as deprived. Among low-income countries, the median proportion of deprivation was 41%, with Chad recording the highest weighted proportion at 74%. Among lower-middle-income countries and territories, the median proportion of deprivation was 11%, ranging from a weighted proportion of 0% in Armenia and Kyrgyzstan to 40% in Mauritania. For upper-middle-income countries and territories, the distribution differs considerably with median deprivation rates of less than 1%. Only in Angola and Peru, the proportions of the non-deprived populations were less than 70%. Comparing all countries, the weighted proportion of vulnerable individuals ranges from less than 1% in Turkmenistan to 66% in Kiribati.

**Table 3:** Description of the surveys included in the analysis of socioeconomic deprivation status, 2015–2019

Country or territory	Survey year	Source	Sufficient sample size for analysis^a^	Income group^b^
Afghanistan	2015	DHS	Yes	Low income
Algeria	2018	MICS	Yes	Upper-middle income
Angola	2015	DHS	Yes	Upper-middle income
Armenia	2015	DHS	No	Lower-middle income
Bangladesh	2019	MICS	Yes	Lower-middle income
Belarus	2019	MICS	No	Upper-middle income
Belize	2015	MICS	No	Upper-middle income
Benin	2017	DHS	Yes	Low income
Burundi	2016	DHS	Yes	Low income
Cameroon	2018	DHS	Yes	Lower-middle income
Central African Republic	2018	MICS	Yes	Low income
Chad	2019	MICS	Yes	Low income
Côte d’Ivoire	2016	MICS	Yes	Lower-middle income
Cuba	2019	MICS	No	Upper-middle income
Democratic Republic of the Congo	2017	MICS	Yes	Low income
Dominican Republic	2019	MICS	Yes	Upper-middle income
Ethiopia	2019	DHS	Yes	Low income
Gambia	2019	DHS	Yes	Low income
Georgia	2018	MICS	No	Upper-middle income
Ghana	2017	MICS	Yes	Lower-middle income
Guinea	2018	DHS	Yes	Low income
Guinea-Bissau	2018	MICS	Yes	Low income
Guyana	2019	MICS	No	Upper-middle income
Haiti	2016	DHS	Yes	Low income
Honduras	2019	MICS	Yes	Lower-middle income
India	2015	DHS	Yes	Lower-middle income
Indonesia	2017	DHS	Yes	Lower-middle income
Iraq	2018	MICS	Yes	Upper-middle income
Kazakhstan	2015	MICS	No	Upper-middle income
Kiribati	2018	MICS	Yes	Lower-middle income
Kosovo	2019	MICS	No	Upper-middle income
Kyrgyzstan	2018	MICS	No	Lower-middle income
Lao People's Democratic Republic	2017	MICS	Yes	Lower-middle income
Liberia	2019	DHS	Yes	Low income
Madagascar	2018	MICS	Yes	Low income
Malawi	2019	MICS	Yes	Low income
Maldives	2016	DHS	No	Upper-middle income
Mali	2018	DHS	Yes	Low income
Mauritania	2015	MICS	Yes	Lower-middle income
Mexico	2015	MICS	No	Upper-middle income
Mongolia	2018	MICS	No	Lower-middle income
Montenegro	2018	MICS	No	Upper-middle income
Mozambique	2015	DHS	Yes	Low income
Myanmar	2015	DHS	Yes	Lower-middle income
Nepal	2019	MICS	Yes	Lower-middle income
Nigeria	2018	DHS	Yes	Lower-middle income
North Macedonia	2018	MICS	No	Upper-middle income
Pakistan	2017	DHS	Yes	Lower-middle income
Papua New Guinea	2016	DHS	Yes	Lower-middle income
Paraguay	2016	MICS	Yes	Upper-middle income
Peru	2020	ENDES	Yes	Upper-middle income
Philippines	2017	DHS	Yes	Lower-middle income
Rwanda	2019	DHS	Yes	Low income
Samoa	2019	MICS	No	Upper-middle income
Sao Tome and Principe	2019	MICS	Yes	Lower-middle income
Senegal	2019	DHS	Yes	Lower-middle income
Serbia	2019	MICS	No	Upper-middle income
Sierra Leone	2019	DHS	Yes	Low income
South Africa	2016	DHS	No	Upper-middle income
Suriname	2018	MICS	No	Upper-middle income
Tajikistan	2017	DHS	No	Low income
Thailand	2019	MICS	No	Upper-middle income
Timor-Leste	2016	DHS	Yes	Lower-middle income
Togo	2017	MICS	Yes	Low income
Tonga	2019	MICS	No	Upper-middle income
Tunisia	2018	MICS	No	Lower-middle income
Turkmenistan	2015	MICS	No	Upper-middle income
Tuvalu	2019	MICS	No	Upper-middle income
Uganda	2016	DHS	Yes	Low income
United Republic of Tanzania	2015	DHS	Yes	Low income
West Bank and Gaza Strip	2019	MICS	No	Lower-middle income
Zambia	2018	DHS	Yes	Lower-middle income
Zimbabwe	2019	MICS	Yes	Lower-middle income

DHS: Demographic and Health Survey; ENDES: *Encuesta Demográfica y de Salud Familiar*; MICS: Multiple Indicator Cluster Survey.

^a^ We did not conduct coverage analyses for a survey if the sample size for any of the three socioeconomic deprivation status measures was less than 25 households.

^b^ We used the World Bank country income level classification^15^ for the year the survey was conducted.

**Fig. 2:**
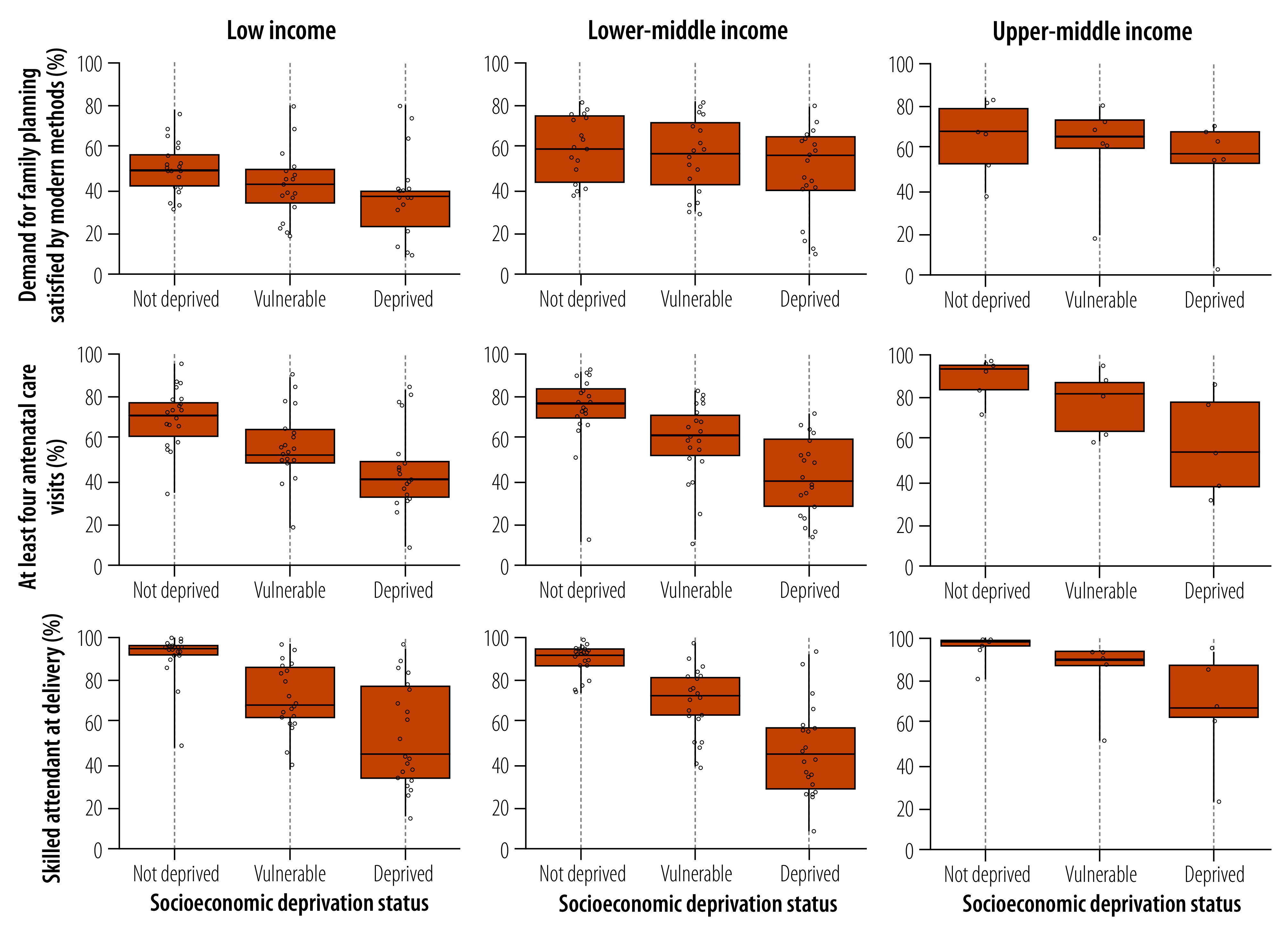
Distribution of reproductive and maternal health indicators by socioeconomic deprivation status, 48 low- and middle-income countries

Note: The whiskers in the box plot show maximum and minimum values, the horizontal lines represent the medians and boxes show interquartile ranges.

In our coverage analysis of reproductive and maternal health interventions, we excluded 25 surveys due to insufficient sample sizes in the deprived group, analysing in total 48 countries. Fig. 2 presents the estimated distribution of the three indicators of reproductive and maternal health for each group of the socioeconomic deprivation status. For all three indicators, the median coverage was consistently lower among individuals in deprived households, regardless of the country income classification. Low-income countries showed a linear equity pattern in the demand for family planning satisfied with modern methods across the different socioeconomic deprivation status groups, showing a median gap of 13 percentage points between deprived and non-deprived groups (Fig. 2). These differences were smaller in lower-middle and upper-middle-income countries, although coverage was still lower among the most deprived individuals. As for indicators on women who received antenatal care in at least four visits, and had a skilled attendant at delivery, the median gaps between groups were much larger than for demand for family planning satisfied with modern methods (Fig. 2). While the median coverage for skilled attendant at delivery for the non-deprived surpassed 90% in all country income categories, the coverage remained below 50% among the deprived households in low- and lower-middle-income countries and was slightly above 60% for upper-middle-income countries.

**Fig. 3:**
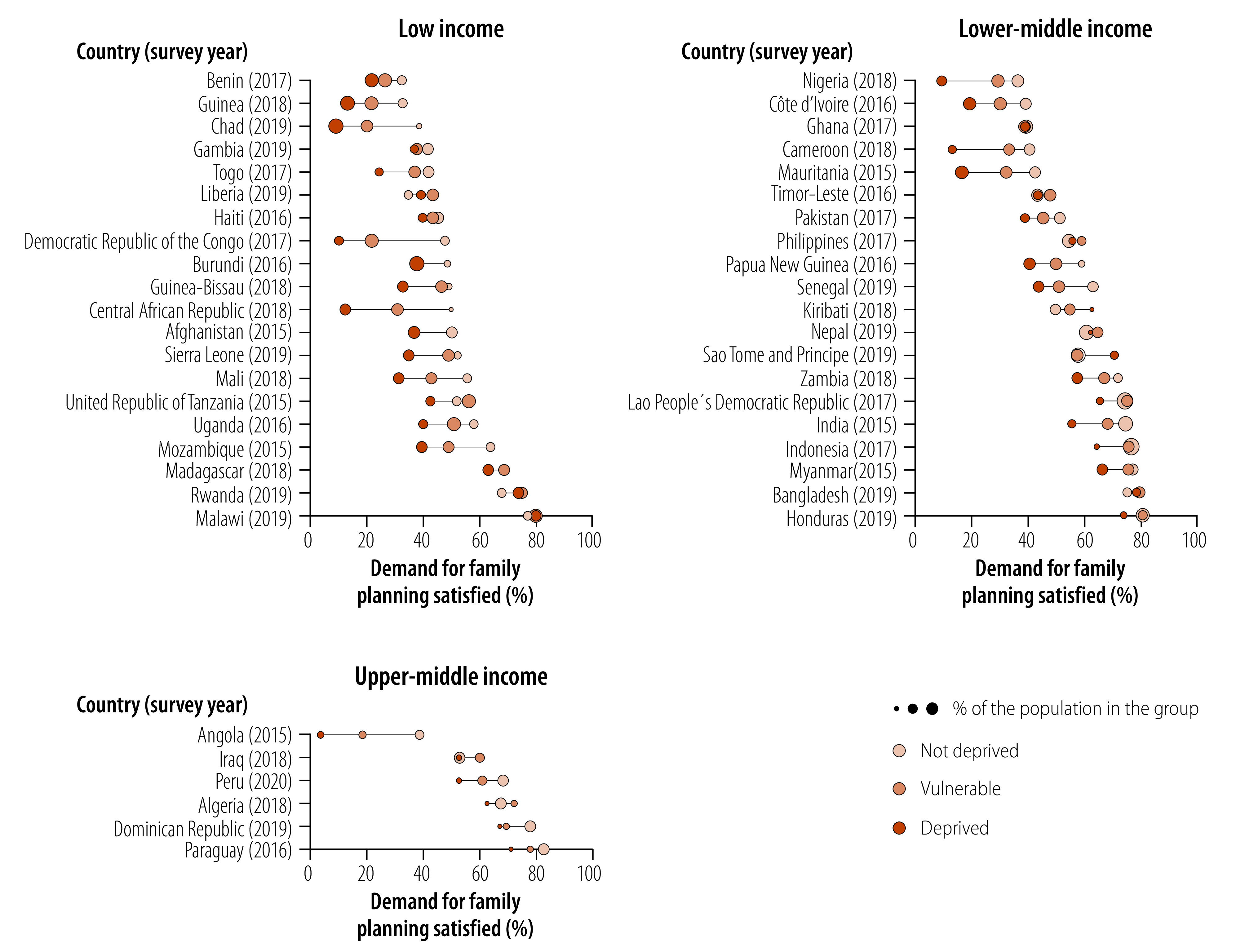
Coverage of demand for family planning satisfied with modern methods by socioeconomic deprivation status, 45 low- and middle-income countries

Sufficient data on demand for family planning satisfied with modern methods were available for 45 countries. Coverage of demand for family planning satisfied with modern methods by country is presented in Fig. 3. Half (nine) of the low-income countries exhibit top inequality and linear inequality patterns, while the remaining half present small differences between the socioeconomic deprivation status groups. We observed more consistent bottom inequality patterns in lower-middle-income countries, where coverage among the deprived is half that of the non-deprived and vulnerable, as seen in Cameroon and Nigeria. Across all 45 countries, the slope index of inequality shows gaps that exceed 20 percentage points in 16 countries, and four countries show higher demand for family planning satisfied with modern methods coverage among the deprived (available in online repository).^16^

**Fig. 4:**
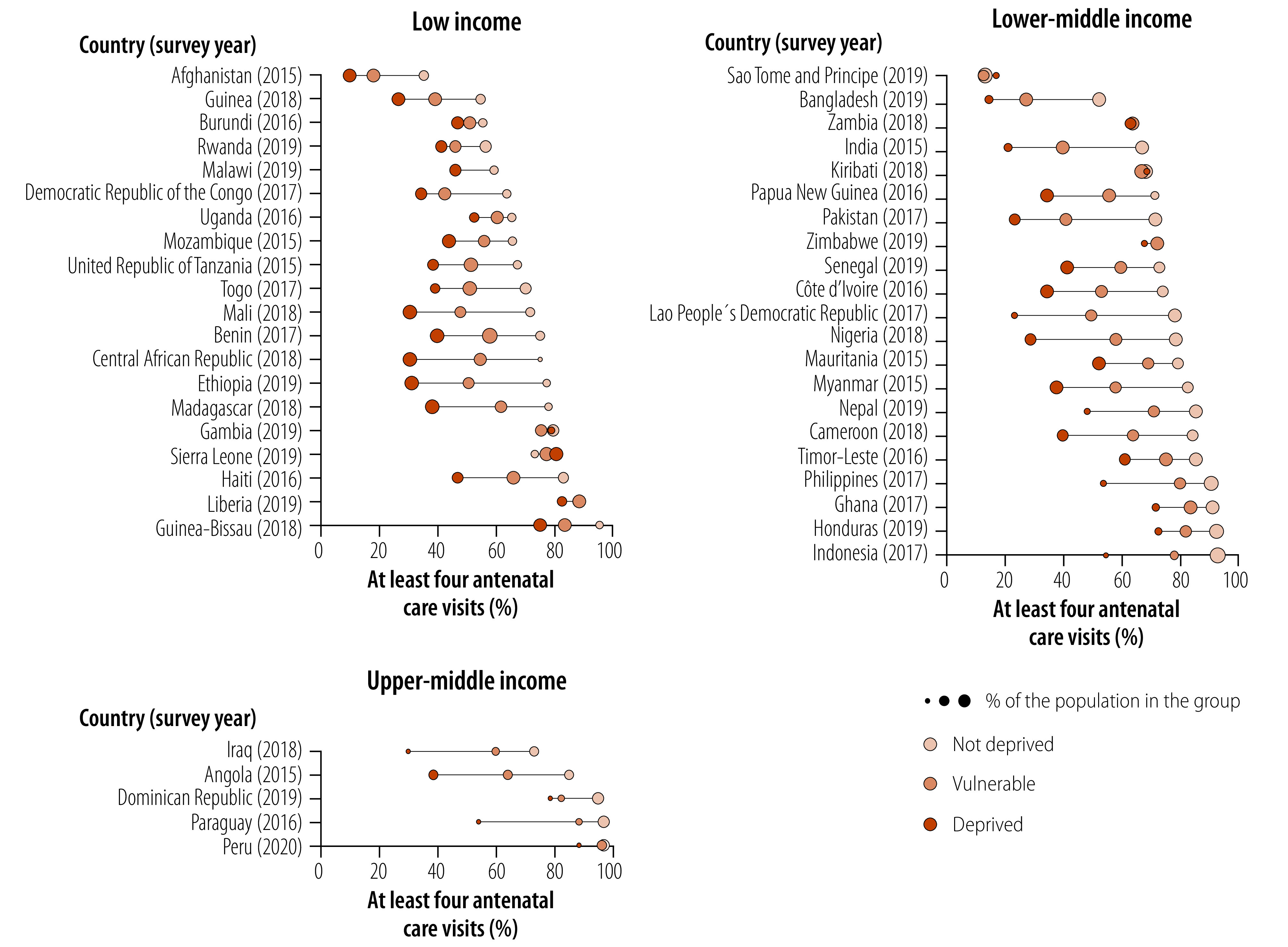
Coverage of women who received antenatal care in at least four visits by socioeconomic deprivation status, 46 low- and middle-income countries

We observed a similar pattern driven by top and linear inequality for women who received antenatal care in at least four visits in low-income countries (Fig. 4). A linear pattern is also evident in lower-middle- and upper-middle-income countries, with few bottom inequality cases such as Indonesia, Iraq, Paraguay and Peru. The magnitude of the differences is much greater for women who received antenatal care in at least four visits in comparison to the demand for family planning satisfied with modern methods. The slope index of inequality shows that gaps above 60 percentage points exist in Angola, Lao People's Democratic Republic, Nigeria and Pakistan (available in online repository).^16^ Only six countries (Gambia, Kiribati, Peru, Sao Tome and Principe, Zambia and Zimbabwe) showed no inequalities between socioeconomic deprivation status groups.

**Fig. 5:**
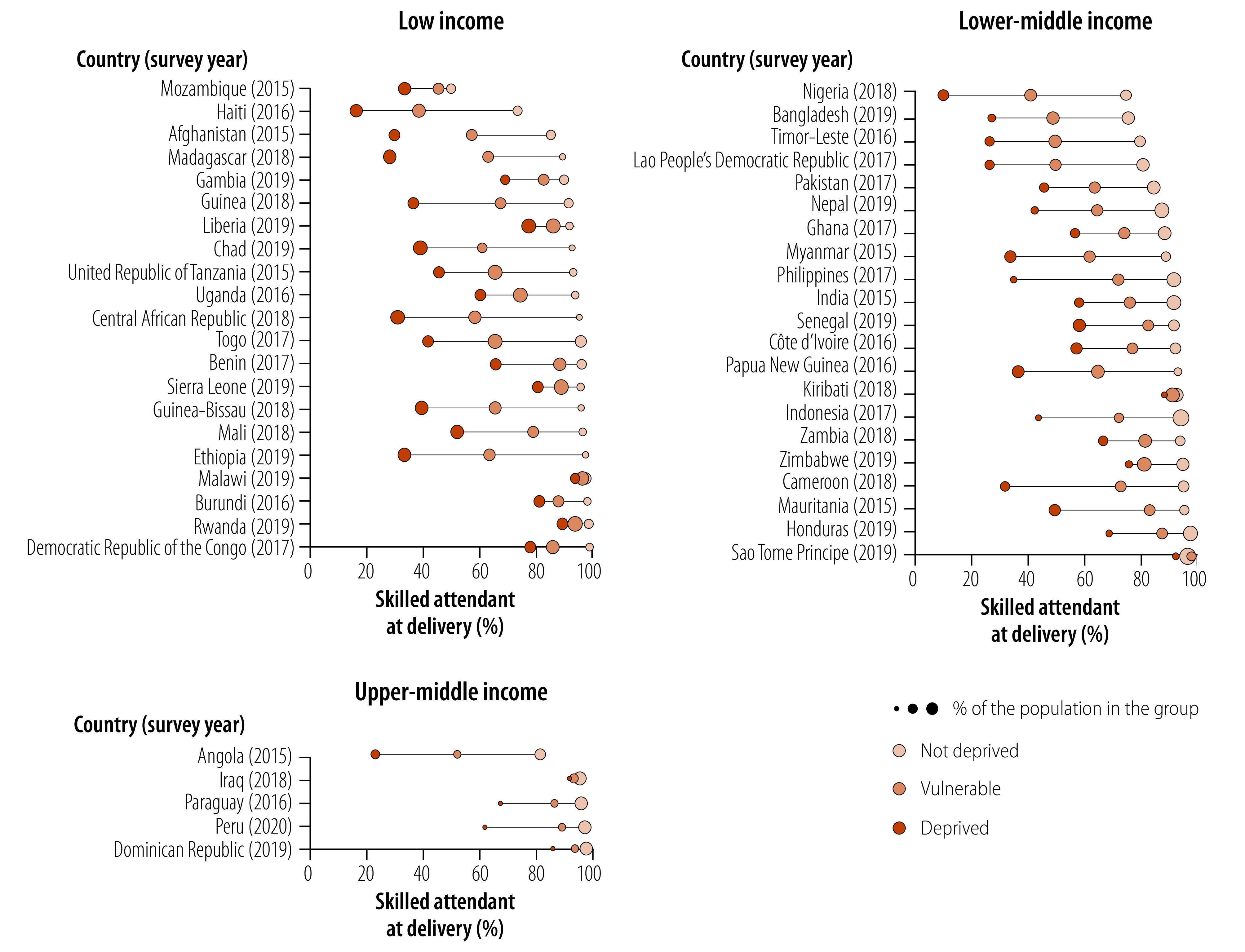
Coverage of skilled attendant at delivery by socioeconomic deprivation status, 47 low- and middle-income countries

Fig. 5 shows that in most countries, individuals in the non-deprived group have considerably higher coverage of skilled attendants at delivery. However, there are widespread gaps between this group and the vulnerable and deprived groups. In some countries, bottom inequality patterns emerge because individuals in the vulnerable group also have almost universal coverage. The slope index of inequality shows that about 80% of countries have gaps above 20 percentage points in coverage of skilled attendant at delivery; and Angola, Cameroon and Nigeria having gaps that surpass 75 percentage points, all in favour of those with less deprivation (available in online repository).^16^

## Discussion

Here we provide a large-scale empirical analysis of reproductive and maternal health inequalities using a novel, absolute and internationally comparable composite index of socioeconomic deprivation status, which is based on household survey data. The results demonstrate a clear contrast in health indicator coverage between deprived or vulnerable individuals and those in more privileged socioeconomic positions. Indeed, findings highlight that reproductive and maternal health coverage among socioeconomically deprived individuals is substantially lower than coverage among privileged individuals in almost all low- and middle-income countries and across the three indicators studied.

Our results are consistent with previous research, which shows that individuals in higher socioeconomic positions are more likely to have a skilled attendant at delivery compared to those facing socioeconomic disadvantages.^17^^,^^18^ Our results indicate that in nearly all countries, least deprived groups are closest to achieving universal coverage. Conversely, in countries such as Haiti and Nigeria, less than one fifth of births among populations living in the poorest socioeconomic settings are attended by skilled professionals. In these countries, which have high urbanization rates and low education levels for women, people with low socioeconomic status may often live in precarious circumstances, including informal settlements.^19^^,^^20^ Financial limitations, barriers to accessing health facilities, and a lack of infrastructure to accommodate the growing population in cities often lead to births occurring without a skilled attendant.^21^^,^^22^ In addition, the rural populations in these countries, which are disproportionately socioeconomically disadvantaged, frequently experience barriers to accessing health care and long distances to facilities.^6^^,^^8^^,^^23^ On the other hand, countries such as Iraq, Malawi and Sao Tome and Principe have managed to achieve high coverage of skilled attendants at delivery across the population. Malawi, for example, released policy guidelines in 2007 which promote skilled attendants at delivery, and prohibit traditional birth attendants from performing routine deliveries.^24^

We also found wide gaps between the socioeconomically deprived group and the less deprived group for women who received antenatal care in at least four visits, and demand for family planning satisfied with modern methods. Similar findings have been published when comparing the extremes of wealth distribution.^25^^–^^27^ Unlike the almost universal access to skilled attendants at delivery among socioeconomically advantaged groups, improvement of coverage in the other two indicators is necessary across all socioeconomic deprivation status groups in the majority of the countries studied. Hence, efforts to reduce inequalities must be planned alongside actions to improve coverage for the entire population. Commitments to boost geographical accessibility, promote women’s education, and implement policies attentive to the needs and desires of the underserved groups are key to advance both these indicators towards universal health coverage (UHC).^28^^,^^29^

The inequality patterns we observed, especially for women who received antenatal care in at least four visits and the presence of skilled attendants at delivery, are aligned with what would be expected according to the inverse equity hypothesis,^13^ that is, when coverage is low, top inequality patterns emerge, and when coverage increases the pattern changes to bottom inequality. Given that median coverage is above 50% for all indicators, linear and bottom inequality patterns are more pronounced in most countries. Equity-oriented policies are necessary to increase coverage in the socioeconomically disadvantaged population, particularly for indicators with greater coverage gaps between groups.

The use of the socioeconomic deprivation status is not without its limitations. First, household surveys may not sample some of the most vulnerable populations, for example, those who are internally displaced or affected by disasters and conflicts. Second, cross-country comparisons are affected by the year of the survey due to the variations in survey periodicity, which also influenced the selection of countries for our analysis. Finally, as an absolute measure, the socioeconomic deprivation status leverages on its fixed cut-offs and clear definition contributing to a more consistent and comparable assessment of socioeconomic inequalities across settings. However, its usefulness is limited in countries with very low levels of thus-defined socioeconomic deprivation, as observed especially among upper-middle-income countries. This limitation is because the socioeconomic deprivation status' design is based on the UN’s global Multidimensional Poverty Index, which intends to measure acute poverty.^8^ Future research may explore an extension to the original socioeconomic deprivation status cut-offs along the lines of a moderate poverty measure, which could be more relevant for higher-income countries and other countries progressing to reduce levels of socioeconomic deprivation.^30^

Our study shows that the socioeconomic deprivation status, as a composite index of absolute socioeconomic deprivation, is suitable to monitor health inequalities through national household survey data.^6^ This composite index can differentiate between groups of households in unfavourable conditions in most low- and middle-income countries. Unlike previous indices focused on reproductive, maternal and child health inequalities, this measure provides a comprehensive baseline for policy analysis and improves our ability to track progress towards the SDGs. Since the composite index captures absolute disadvantage, it facilitates comparisons across various disaggregated levels and different geographic areas, such as subnational regions, provided that adequate sample sizes are available. Furthermore, the composite index's comparability over time enables the evaluation of the long-term impacts of equity-focused policies. For countries conducting consecutive health surveys, this composite index can become a valuable tool to monitor and analyse trends in health coverage disparities.

In conclusion, the study revealed major gaps in three reproductive and maternal health indicators mostly favouring the socioeconomically advantaged. Addressing these inequalities, as well as increasing coverage in many countries, is key to meeting the SDGs, in particular, targets related to UHC and preventing maternal and child mortality, as well as achieving the 2030 sustainable development agenda of leaving no one behind.^1^

## References

[1] Resolution A/RES/70/1. Transforming our world: the 2030 agenda for sustainable development. In: Seventieth United Nations General Assembly, New York, 25 September 2015. New York: United Nations; 2015. Available from: https://undocs.org:443/A/RES/70/1 [cited 2023 Sep 6].

[2] A conceptual framework for action on the social determinants of health. Geneva: World Health Organization; 2010.

[3] Faye CM, Wehrmeister FC, Melesse DY, Mutua MKK, Maïga A, Taylor CM, et al. Large and persistent subnational inequalities in reproductive, maternal, newborn and child health intervention coverage in sub-Saharan Africa. BMJ Glob Health. 2020 Jan 26;5(1):e002232. 10.1136/bmjgh-2019-002232 32133183 PMC7042572

[4] World health statistics 2023: monitoring health for the SDGs, sustainable development goals. Geneva: World Health Organization; 2023. Available from: https://www.who.int/publications/i/item/9789240074323 [cited 2023 Nov 27].

[5] Hosseinpoor AR, Bergen N, Schlotheuber A, Grove J. Measuring health inequalities in the context of sustainable development goals. Bull World Health Organ. 2018 Sep 1;96(9):654–9. 10.2471/BLT.18.210401 30262947 PMC6154075

[6] Global multidimensional poverty Index 2023. Unstacking global poverty: data for high impact action. New York, Oxford: United Nations Development Programme & Oxford Poverty and Human Development Initiative; 2023.

[7] Filmer D, Pritchett LH. Estimating wealth effects without expenditure data–or tears: an application to educational enrollments in states of India. Demography. 2001 Feb;38(1):115–32. 11227840 10.1353/dem.2001.0003

[8] Dirksen J, Pinilla-Roncancio M, Wehrmeister FC, Ferreira LZ, Vidaletti LP, Kirkby K, et al. Exploring the potential for a new measure of socioeconomic deprivation status to monitor health inequality. Int J Equity Health. 2022 Apr 23;21(1):56. 10.1186/s12939-022-01661-0 35461294 PMC9034442

[9] Smits J, Steendijk R. The international wealth index (IWI). Soc Indic Res. 2015 May 12;122(1):65–85. 10.1007/s11205-014-0683-x

[10] Martel P, Mbofana F, Cousens S. The polychoric dual-component wealth index as an alternative to the DHS index: addressing the urban bias. J Glob Health. 2021 Jan 30;11:04003. 10.7189/jogh.11.04003 33643634 PMC7897450

[11] Corsi DJ, Neuman M, Finlay JE, Subramanian SV. Demographic and health surveys: a profile. Int J Epidemiol. 2012 Dec;41(6):1602–13. 10.1093/ije/dys184 23148108

[12] Murray C, Newby H. Data resource profile: United Nations Children’s Fund (UNICEF). Int J Epidemiol. 2012 Dec;41(6):1595–601. 10.1093/ije/dys185 23211414 PMC3535745

[13] Victora CG, Joseph G, Silva ICM, Maia FS, Vaughan JP, Barros FC, et al. The inverse equity hypothesis: analyses of institutional deliveries in 286 national surveys. Am J Public Health. 2018 Apr;108(4):464–71. 10.2105/AJPH.2017.304277 29470118 PMC5844402

[14] Schlotheuber A, Hosseinpoor AR. Summary measures of health inequality: a review of existing measures and their application. Int J Environ Res Public Health. 2022 Mar 20;19(6):3697. 10.3390/ijerph19063697 35329383 PMC8992138

[15] Fantom N, Serajuddin U. The World Bank’s Classification of Countries by Income. Washington, DC: World Bank; 2016. Available from: https://openknowledge.worldbank.org/handle/10986/23628 [cited 2023 Sep 6].

[16] Ferreira LZ, Wehrmeister FC, Dirksen J, Vidaletti LP, Pinilla-Roncancio M, Kirkby K, et al. Supplemental information for a novel inequality dimension for socioeconomic deprivation applied to survey data on reproductive and maternal health coverage. [online repository]. Charlottesville: Center of Open Science; 2023. 10.17605/OSF.IO/3DUSM

[17] Joseph G, da Silva ICM, Wehrmeister FC, Barros AJD, Victora CG. Inequalities in the coverage of place of delivery and skilled birth attendance: analyses of cross-sectional surveys in 80 low and middle-income countries. Reprod Health. 2016 Jun 17;13(1):77. 10.1186/s12978-016-0192-2 27316970 PMC4912761

[18] Wong KLM, Restrepo-Méndez MC, Barros AJD, Victora CG. Socioeconomic inequalities in skilled birth attendance and child stunting in selected low and middle income countries: Wealth quintiles or deciles? PLoS One. 2017 May 3;12(5):e0174823. 10.1371/journal.pone.0174823 28467411 PMC5414946

[19] Ooi GL, Phua KH. Urbanization and slum formation. J Urban Health. 2007 May;84(3) Suppl 1:27–34. 10.1007/s11524-007-9167-5 17387618 PMC1891640

[20] Kruk ME, Prescott MR, Galea S. Equity of skilled birth attendant utilization in developing countries: financing and policy determinants. Am J Public Health. 2008 Jan;98(1):142–7. 10.2105/AJPH.2006.104265 18048785 PMC2156044

[21] Yaya S, Bishwajit G, Uthman OA, Amouzou A. Why some women fail to give birth at health facilities: a comparative study between Ethiopia and Nigeria. PLoS One. 2018 May 3;13(5):e0196896. 10.1371/journal.pone.0196896 29723253 PMC5933759

[22] Metcalfe R, Adegoke AA. Strategies to increase facility-based skilled birth attendance in South Asia: a literature review. Int Health. 2013 Jun;5(2):96–105. 10.1093/inthealth/ihs001 24030109

[23] Tackling inequalities in public service coverage to “build forward better” for the rural poor. Policy brief by the HLCP inequalities task team. New York: United Nations; 2021.

[24] Cammack D. Local governance and public goods in Malawi. IDS Bull. 2011 Mar;42(2):43–52. 10.1111/j.1759-5436.2011.00210.x

[25] McKinnon B, Harper S, Kaufman JS. Do socioeconomic inequalities in neonatal mortality reflect inequalities in coverage of maternal health services? Evidence from 48 low- and middle-income countries. Matern Child Health J. 2016 Feb;20(2):434–46. 10.1007/s10995-015-1841-8 26546016

[26] Hellwig F, Coll CVN, Blumenberg C, Ewerling F, Kabiru CW, Barros AJD. Assessing wealth-related inequalities in demand for family planning satisfied in 43 African countries. Front Glob Womens Health. 2021 Jul 26;2:674227. 10.3389/fgwh.2021.674227 34816227 PMC8594043

[27] Anindya K, Marthias T, Vellakkal S, Carvalho N, Atun R, Morgan A, et al. Socioeconomic inequalities in effective service coverage for reproductive, maternal, newborn, and child health: a comparative analysis of 39 low-income and middle-income countries. EClinicalMedicine. 2021 Sep 7;40:101103. 10.1016/j.eclinm.2021.101103 34527893 PMC8430373

[28] Okedo-Alex IN, Akamike IC, Ezeanosike OB, Uneke CJ. Determinants of antenatal care utilisation in sub-Saharan Africa: a systematic review. BMJ Open. 2019 Oct 7;9(10):e031890. 10.1136/bmjopen-2019-031890 31594900 PMC6797296

[29] Hellwig F, Barros AJ. Learning from success cases: ecological analysis of potential pathways to universal access to family planning care in low- and middle-income countries. Gates Open Res. 2023 Jan 20;6:59. 10.12688/gatesopenres.13570.3 36726686 PMC9873636

[30] Alkire S, Kövesdi F, Scheja E, Vollmer F. Moderate multidimensional poverty index: paving the way out of poverty. Soc Indic Res. 2023 May 27;168:409–45. 10.1007/s11205-023-03134-5 37362183 PMC10220327

